# Self-Efficacy to Engage in Physical Exercise and Walking Ability Best Predicted Exercise Adherence after Stroke

**DOI:** 10.1155/2020/2957623

**Published:** 2020-03-04

**Authors:** Lívia C. G. Caetano, Bruna D. Pacheco, Giane A. R. Samora, Luci F. Teixeira-Salmela, Aline A. Scianni

**Affiliations:** Graduate Program in Rehabilitation Sciences, Department of Physical Therapy, Universidade Federal de Minas Gerais, Belo Horizonte 31270-901, Brazil

## Abstract

**Aim:**

To describe exercise preferences and to investigate the contribution of exercise preferences, walking ability, and current levels of physical activity in predicting exercise adherence in individuals with chronic stroke.

**Methods:**

For this exploratory study, exercise adherence was measured using the first question of the first section of the Exercise Preference Questionnaire (stroke)-Brazil (EPQ (stroke)-Brazil). Nine independent variables were included as potential predictors of exercise adherence: the seven factors of the EPQ (stroke)-Brazil, walking speed, and level of physical activity.

**Results:**

Participated 93 individuals with stroke, who had a mean age of 62 (SD 12) years and a mean time since the onset of the stroke of 58 (SD 67) months. The most preferable exercise was walking. Logistic regression analysis revealed that self-efficacy to engage in physical exercise and walking ability predicted and explained 80% of the variance in exercise adherence.

**Conclusion:**

The findings showed that feeling able to perform physical exercise and having higher walking ability predicted higher exercise adherences in individuals with chronic stroke. The knowledge of potential contributors to exercise adherence may help in designing exercise programs for individuals with stroke.

## 1. Introduction

Stroke is the major cause of death and long-term disability in the world [1]. Among the major modifiable risk factors for the occurrence and recurrence of stroke, physical inactivity is considered the most important [1, 2].

Although the terms physical activity and exercise are usually interchangeably used, they have different meanings. Physical activity refers to any bodily movements produced by the contractions of the skeletal muscles that increase energy expenditure, such as those executed during leisure and work activities. Exercise is a subcategory of physical activity, which consists of a planned, structured, and repetitive physical activity aimed at maintaining or improving physical fitness. Clinical guidelines advise adults to perform at least 30 minutes of moderate-intensity exercises, five times a week, for at least 150 minutes per week [3, 4].

It has been reported that about 77% of stroke survivors are sedentary or have low levels of physical activity [4]. This can be explained by decreased movement speed, mainly observed in individuals with severe motor impairments [5]. However, even stroke survivors with mild motor impairments, who are community walkers and able to engage in community-based exercises, show low levels of physical activity [6]. This suggests that environmental and personal factors may also act as barriers to exercise [7–9].

The understanding of the predictors of exercise adherence, as well as the identification of exercise preferences, may allow rehabilitation professionals to design more appropriate exercise programs, thus, leading to more effective results. Studies, which investigated factors that could prevent stroke survivors from engaging in exercise, found that fear of falling, difficulties in accessing exercise venues, high costs, transportation issues, fatigue, lack of motivation, lack of a partner, depressive symptoms, and motor impairments due to the stroke itself, were the main reported barriers [8, 10–12]. The results of a single study which investigated exercise preferences after stroke in Australia [13] showed that individuals with stroke prefer to perform structured exercises in groups and in places, such as gyms and community centers [13]. In addition, lower activity levels were found to be associated with higher preferences for structured environments [13].

The findings of the abovementioned study may not reflect exercise preferences in individuals with stroke living in different regions of the world. In Brazil, which is a low-income country, exercise preferences of individuals with stroke have not been identified. To our knowledge, this is the first study to investigate together the contribution of exercise preferences, such as presence of instructions/planning, program flexibility, and exercise in group/alone, to exercise adherence after stroke.

Therefore, the objectives of the present study were to describe exercise preferences and investigate the contribution of exercise preferences, walking ability, and current levels of physical activity in predicting exercise adherence in individuals with chronic stroke living in Brazil. The findings may help professionals to design strategies to increase exercise adherence, in order to improve health outcomes.

## 2. Materials and Methods

### 2.1. Study Design and Participants

For this cross-sectional, exploratory study, the contacts of individuals with stroke were obtained from the lists of previous research projects and referral from the health professionals of public stroke health units of the city of Belo Horizonte, Brazil. To be included, the participants should have clinical diagnosis of chronic stroke, i.e., more than six months after the onset of a hemorrhagic or an ischemic stroke, confirmed by their medical charts, ≥20 years of age, the ability to walk 5 meters independently with or without walking aids, and no cognitive deficits, as determined by the following education-adjusted scores on the mini-mental state examination: 13 for the individuals with illiteracy, 18 for those with basic education, and 26 for those with high education [14].

### 2.2. Procedures

Initially, the participants were informed about the objectives of the study and were invited to provide written consent, based upon previous approval from both the institutional research ethical committee and the healthcare city secretariat (#34921814.8.0000.5149).

Then, demographic and clinical data, such as age, sex, and time since the onset of the stroke, as well as self-rated general health (excellent, very good, good, fair, or poor), which was assessed using the patient-reported outcome measurement information system (PROMIS) [15], were collected for characterization purposes, followed by the collection of the outcome measures (exercise preferences, walking speed, and level of physical activity). Data were collected on one day within a laboratory setting by trained research personnel, who had clinical and research experiences in the area of stroke rehabilitation. All self-reported data were collected by means of interviews.

### 2.3. Outcome Measures

#### 2.3.1. Independent Variables

The following independent variables were included, as potential predictors of exercise adherence: exercise preferences, walking speed, and level of physical activity.

Exercise preferences were identified by the Exercise Preference Questionnaire (stroke)-Brazil (EPQ (stroke)-Brazil), which has shown to be a valid and reliable measure to be used with individuals with stroke [16]. It consists of 33 questions, which are divided into three sections. The first section contains three items that identify if the individual is currently participating in an organized exercise program, the frequency, and the type of physical exercise. The second section consists of 22 items which assess the individuals' opinions regarding the following factors: (1) presence of instructions/planning, (2) feeling able to perform physical exercises, (3) exercise with family/friends-program flexibility, (4) exercises in fitness centers with people of similar age, (5) exercise alone, (6) exercises in fitness centers with people with stroke, and (7) routine (planned, instructed, gentle, at home, and morning exercises) [16]. Finally, the third section contains five questions (four of which are open) about individual exercise preferences.

The EPQ (stroke)-Brazil does not provide a total score, since the objective is to screen the contextual factors related to the practice of physical exercise [16]. Participants were asked how much they agreed with each of the 22 statements of the second section of the EPQ (stroke)-Brazil on a scale ranging from 0 to 100%. Previous exploratory factor analysis, which was used to identify the factors associated with exercise preferences, revealed that the following seven factors emerged: (1) presence of instructions/planning, (2) ability to perform physical exercises, (3) exercise with family/friends-program flexibility, (4) exercise in fitness centers-people of similar age, (5) exercise alone, (6) exercise in fitness centers-people with stroke, and (7) routine (planned, instructed, gentle, at home, and morning exercises) [16]. Therefore, the participants' responses were grouped into the seven abovementioned factors, and the load of each factor was used as a potential predictor [16].

Walking ability was determined by walking speed, which was measured while the participants walked at their comfortable speeds along a 7 m hallway, wearing their usual shoes and walking devices [17]. The time to cover the central 5 m was recorded with a digital stopwatch and the speed, in m/s, was calculated. The 5 min walk test is responsive to changes in functional capacity and showed adequate test-retest reliability for individuals with stroke [17]. After familiarization, one trial was recorded [17].

General level of physical activity was assessed by the Brazilian version of the human activity profile (HAP) [18, 19], which has adequate psychometric properties and has been widely used for clinical and research purposes with individuals with stroke [19, 20]. The HAP provides a survey of activities across a broad range of energy requirements, including activities from low to high activity levels. The HAP includes 94 activities, such as self-care, transportation, home maintenance, entertainment/social, and physical exercises, which are sequentially rated according to their required metabolic equivalents, so that a score of 1 represents the lowest and 94 the highest metabolic equivalent value. For example, item number 1 corresponds to getting in and out of chairs or bed without help, whereas item number 94 corresponds to running or jogging 3 miles in 30 minutes or less [18].

For each item, there are three possible responses: “still doing the activity,” “have stopped doing the activity,” and “never did the activity.” The administration and scoring procedures followed recommended protocols, and scores were tallied to provide a maximum activity score, which indicated the highest metabolic equivalent activity level at which the subject could still perform. An adjusted activity score was determined by subtracting the number of activities that the respondent had discontinued performing from the maximum score and indicated the average typical metabolic equivalent levels. Coefficients for test-retest reliability have been reported at 0.94 for the maximum and 0.79 for the adjusted activity scores [19]. Based upon the AAS values, the individuals were classified as impaired (AAS < 53), moderately active (53 < AAS < 74), or active (AAS > 74) [20].

#### 2.3.2. Dependent Variable

Exercise adherence was measured using the first question of the first section of the EPQ (stroke)-Brazil [16]. The participants were asked to answer the following question: “Do you currently participate in an organized exercise program?” If their answers were positive, they were classified as physical exercise practitioners and if their answers were negative, they were classified as nonpractitioners.

#### 2.3.3. Sample Size

The sample size was calculated to include 10 participants per independent variable [21, 22]. Therefore, the least required number of participants would be 90.

### 2.4. Statistical Analysis

Descriptive statistics, tests for normality (Kolmogorov-Smirnov), and equality of variances (Levene) were calculated for all outcomes. Point biserial correlation analyses were employed to investigate the associations between the potential predictors and exercise adherence. Those variables which, in the biserial correlation analysis, had a *p* value up to 0.10 were included in the logistic regression analysis using the forward stepwise likelihood ratio method. The level of significance was set at *p* < 0.10, to ensure that potential predictors were not excluded at this stage. The final regression model included only the variables which showed significant correlations with exercise adherence, i.e., feeling able to perform physical exercises (EPQ (stroke)-Brazil-Factor 2), walking speed, and level of physical activity (AAS). The significance level was set at *p* < 0.05. Adjustment of the final logistic model was performed by the Hosmer-Lemeshow test and residual analysis. All analyses were carried out using the SPSS for Windows software (version 21.0), at a significance level of 5% [23].

## 3. Results

### 3.1. Flow of the Participants

From a list of 235 potential individuals, 114 were excluded due to refusals (39), incorrect contact information (37), inability to communicate (5), <6 months after the onset of the stroke (14), other associated health conditions (13), cognitive impairments (5), and death (1). Out of the 121, who were eligible and agreed to participate, 28 did not show up for the measurement session. Therefore, 93 individuals were included.

### 3.2. Participants' Characteristics

Ninety-three individuals, 42 males who had a mean age of 62 years (SD 12) and a mean time since the onset of the stroke of 58 months (SD 67), participated. The majority had ischemic stroke (54%), low educational levels (<8 years of schooling) (67%), and reported good to excellent health (58%). Eleven (12%) individuals used walking aids. Their mean AAS score was 60 (SD 15), and 53 individuals were classified as moderately active. Out of the 93 participants, 62 (67%) reported being involved in an organized exercise program and therefore were classified as physical exercise practitioners. Their characteristics are reported in Table 1.

### 3.3. Participants' Preferences

Forty-nine participants (53%) reported walking as their preferred exercise. Regarding the organization of exercise programs, 75 (80%) participants agreed that they liked someone to demonstrate how exercise should be performed, to work hard during exercise, and sessions to be planned; 73 (78%) agreed that they liked to have someone else to organize their exercise sessions, whereas 85 (91%) believed that exercise helps to prevent stroke recurrence.

### 3.4. Prediction of Exercise Adherence

The correlation coefficients between exercise adherence and all measured predictors are displayed in Table 2. Statistically significant associations were found between exercise adherence and feeling able to perform physical exercises (EPQ (stroke)-Brazil-Factor 2), walking speed, and level of physical activity (AAS). Therefore, they were included in the logistic regression analysis; however, level of physical activity was not retained in the model (OR 0.96, *p* = 0.33).

The regression model revealed that self-efficacy, i.e., feeling able to exercise (OR 7; 95% CI 2.8-17.5) and walking speed (OR 56; 95% CI 5-617) predicted and explained 80% of the variance in exercise adherence. These results indicated that feeling able to perform physical exercises and having higher walking ability significantly predicted exercise adherence (Table 3).

## 4. Discussion

This study aimed at describing exercise preferences and investigating the contribution of exercise preferences, walking ability, and levels of physical activity in predicting exercise adherence in individuals with chronic stroke. The findings showed that the majority of the participants preferred someone else to organize, plan, and demonstrate how exercise should be performed, suggesting poor self-efficacy or some dependency on others to design and conduct their exercise program. The regression analysis showed that feeling able to perform exercise and having higher walking abilities significantly predicted exercise adherence. These results confirmed our hypothesis that, in addition to physical factors, environmental and personal factors may also act as barriers to exercise.

The present results corroborate those of the previous studies which investigated barriers to exercise after stroke [8, 9, 11, 24], although they did not examine physical, environmental, and personal factors together in their analyses. A previous study [9] found that measures of walking ability, including walking speed and distance, were correlated with levels of physical activity after stroke. Fini et al. [25] investigated the relationships between walking speed and practice of physical activity, according to the participants' walking speed categories. It was found that the participants who walked at speeds ≤ 0.8/m/s performed less moderate to vigorous physical activity and had more sedentary time than those who walked faster, i.e., >08 m/s. They argued that although the findings provided important information to support the implementation of strategies to manage recovery of physical fitness after stroke, relevant variables such as motivation and self-efficacy were not measured [25].

The results from a survey-based study [24] showed that self-efficacy and outcome expectations regarding exercise influenced self-reported exercise behavior after stroke. Additionally, fear of falling, functional mobility limitations, and negative beliefs regarding physical activity were reported to be the main barriers to maintain sufficient physical activity levels after stroke [11].

In the present study, the majority of the participants reported walking as the mostly preferred exercise and had walking speeds compatible with community walking. Considering that physical activity levels after stroke are associated with walking speed [26, 27], gait training should be early implemented to increase physical activity levels and engagement in exercise programs. However, a recent study with individuals with stroke with similar characteristics in Brazil showed that lack of places to exercise was reported as the second most important barrier, followed by long distances from exercise venues [8]. Guidance on the available locations for exercise near their homes should be provided by health professionals. Although walking practice does not require equipment and structured environment, it requires accessible environment with even sidewalks, which is not the case of the city of Belo Horizonte, Brazil. Public policy should focus on the construction and/or reconstruction of walking lanes, to favor the practice of exercise by individuals with motor disabilities.

In addition, it seems that self-efficacy is an important requirement for people with stroke to engage in exercise. Self-efficacy has been defined as people's beliefs about their capabilities to produce designated levels of performance [28]. It can determine how people feel, think, motivate themselves, and behave with regard to their health. Dixon et al. [29] reported that self-efficacy influences motivation to participate in neurological rehabilitation among adults with neurological diseases [29]. Self-management programs have been suggested as strategies to empower and encourage people, primarily through the expansion of skills, such as problem-solving and decision-making, therefore building self-efficacy to alter long-term behaviors [30]. There is some evidence that self-management programs can result in better long-term outcomes after stroke [31, 32].

It should be noted that other factors, which were not addressed in the present study, have shown to be associated with exercise adherence. The results of Pacheco et al. [8] demonstrated that depressive symptoms and socioeconomic status were the main barriers to exercise after mild stroke. Banks et al. [13] investigated the associations between exercise preferences and physical activity levels, quality of life, and psychological well-being. They found that lower current physical activity levels were associated with higher preference for structure and lower preference for independence. Lower quality of life was associated with higher preference for structure and for exercising at a facility.

The present study is not without limitations. First, the independent variables were selected based upon the literature and clinical experience, but not all possible potential predictors were measured or analyzed. For instance, factors, such as fear of falling and depressive symptoms, were not included as potential predictors. Second, although exercise adherence was measured using a validated tool, i.e., the first section of the EPQ (stroke)-Brazil, it does not quantify other exercise parameters, such as frequency and intensity, which could have influenced the results and deserve to be considered in future studies. Finally, the present results should not be generalized to individuals with different characteristics. This study involved a selected group of individuals with stroke at the chronic stages.

## 5. Conclusions

The findings of the present study showed that participants reported walking as their mostly preferable exercise. Self-efficacy, i.e., feeling able to perform exercise, and higher walking ability predicted and explained 80% of the variance in exercise adherence in individuals with chronic stroke. These findings reflect the need of better understanding the relationships between psychological factors and long-term outcomes after stroke. Considering that walking was the mostly preferable exercise, rehabilitation interventions should combine walking training with interventions that target increases in self-efficacy.

## Figures and Tables

**Table 1:** Characteristics of the participants.

Characteristics	*n* = 93
Age (years), mean (SD)	62 (12)
Sex, *n* females (%)	51 (55)
Time since stroke (months), mean (SD)	58 (67)
One stroke episode, *n* (%)	80 (86)
MMSE (scores: 0-30), mean (SD)	26 (3.1)
Walking speed (m/s), mean (SD), and range (min-max)	0.86 (0.40), (0.11-1.79)
Exercise practitioners, *n* (%)	62 (67)
HAP AAS (scores: 0-94), *n* (%)	
Impaired (AAS < 53)	27 (29)
Moderately active (53 < AAS < 74)	53 (57)
Active (>74)	13 (14)
General health, *n* (%)	
Excellent	12 (12.9)
Very good	6 (6.5)
Good	36 (38.7)
Fair	32 (34.4)
Poor	7 (7.5)
Exercise practitioners, *n* (%)	62 (67)

SD = standard deviation; min = minimum; max = maximum; HAP = human activity profile; AAS = adjusted activity score; MMSE = mini-mental state examination.

**Table 2:** Correlation coefficients and statistical significance (*p* values) between exercise adherence and all measured predictors (*n* = 93).

Predictors		Coefficient	*p* value
EPQ (stroke)-Brazil factors	(1) Presence of instruction/planning	0.10	0.34
	(2) Feeling able to exercise	**0.62**	**<0.0001**
	(3) Exercise with family/friends	0.26	0.81
	(4) Exercise in fitness centers with people of same age	0.05	0.67
	(5) Exercise alone	-0.13	0.22
	(6) Exercise in fitness centers with people with stroke	0.01	0.92
	(7) Routine	-0.04	0.68
Walking speed (m/s)		**0.46**	**0.001**
HAP adjusted activity scores		**0.24**	**0.02**

EPQ (stroke)-Brazil = Exercise Preference Questionnaire (stroke)-Brazil; HAP = human activity profile.

**Table 3:** Results of the logistic regression analysis regarding the predictors of exercise adherence after stroke (*n* = 93).

Predictors	*B*	*p* value	OR (95% CI)
Constant	-1.88	—	—
Feeling able to exercise	1.95	<0.0001	7.0 (2.8-17.5)
Walking speed	4.03	0.001	56.3 (5-617)

OR = odds ratio; CI = confidence interval.

## Data Availability

Data may be available upon request to the corresponding author.
